# A Comparative Prevalence of Birth Defects between Newborns of Immigrant and Native-Born Mothers in Taiwan: Ten Years of Population-Based Data

**DOI:** 10.3390/ijerph182312530

**Published:** 2021-11-28

**Authors:** Yu-Jung Lin, Jeng-Yuan Chiou, Jing-Yang Huang, Pen-Hua Su, Jia-Yuh Chen, Hao-Jan Yang

**Affiliations:** 1 Department of Public Health, Chung Shan Medical University, Taichung 40201, Taiwan; Maureen781211@gmail.com; 2 School of Health Policy and Management, Chung Shan Medical University, Taichung 40201, Taiwan; tom@csmu.edu.tw; 3 Clinical Research Center, Chung Shan Medical University Hospital, Taichung 40201, Taiwan; wchinyang@gmail.com; 4 Institute of Medicine, Chung Shan Medical University, Taichung 40201, Taiwan; jen@csh.org.tw (P.-H.S.); 182288@cch.org.tw (J.-Y.C.); 5 Department of Pediatrics, Chung Shan Medical University Hospital, Taichung 40201, Taiwan; 6 Division of Neonatology, Changhua Christian Children’s Hospital, Changhua 50050, Taiwan; 7 Department of Family and Community Medicine, Chung Shan Medical University Hospital, Taichung 40201, Taiwan

**Keywords:** birth defect, newborns of immigrant mothers, prevalence, stereotype

## Abstract

In recent years, newborns born to immigrant mothers have accounted for about 10% of the total births in Taiwan. However, little is known about whether there are differences between newborns of immigrant and native-born mothers regarding the prevalence and the possible causes of birth defects. By combining four nationwide databases and assessing all newborns between 2005 and 2014 in Taiwan as research subjects, this study determined the prevalence of birth defects stratified into nine categories (neuronal, facial, cleft, circulatory, respiratory, digestive, urogenital, musculoskeletal and chromosomal abnormalities) in the newborns of immigrant mothers and native-born mothers. We found that the prevalence of any birth defects in newborns of immigrant mothers (ranging from 0.98 to 1.24%) was lower than that of native-born mothers (2.86%). Skeletomuscular system defects are the most common among newborns of women from the main immigrant countries (0.24–0.42%), while circulatory system defects were the most common among newborns of Taiwanese women (0.92%). The risks of all defects remained lower for newborns of immigrant mothers (AORs ranged from 0.37 to 0.47) after controlling for possible confounding variables. The higher rates of birth defects among newborns of native-born mothers may be attributed to an older maternal age at childbirth and a higher prevalence of diabetes than that of immigrant mothers. The findings from this study imply that the prevalence of birth defects between newborns of immigrant and native-born mothers is not similar, as evidenced by a decade of population-based data.

## 1. Introduction

Birth defects are complex conditions caused by a variety of genetic and environmental factors and are the main cause of mortality and morbidity in the neonatal period [1,2]. It has been estimated that about 303,000 0–4-week-old newborn infants die each year due to birth defects around the world [3], and one in every five newborn deaths in Taiwan is attributable to birth defects [4]. Birth defects are also one of the five leading causes of death in children and adolescents aged 1–17 in Taiwan [5], and are correlated with childhood and adulthood disorders [6]. For example, normal social interaction skills are affected in children with cleft lips and palates during adolescence due to low self-esteem and timidity [7]. Additionally, psychological functions are also affected [8].

Taiwanese ethnic groups span diverse races and cultures. Since the 1980s, a large number of Southeast Asian women have immigrated to Taiwan through marriage and have contributed a considerable proportion of newborns. From 2005 to 2014, immigrant women gave birth to about one in eight newborns [9]. The different ancestral backgrounds of immigrant women provide clues to genetic and cultural variations for the assessment of the causes of birth defects in newborns. This has facilitated the formulation of preventative policies and austerity within the health care system. A large-scale ethnic survey in the United States indicated that the prevalence of 27 types of birth defects in the Chinese and Asian ethnic groups is lower than that in non-Hispanic Caucasians. However, the risk of newborns with Chinese descent having anotia or microtia and the risk of newborns of Vietnamese descent having Tetralogy of Fallot are higher [10]. These results suggest that ancestral-related factors, such as heredity, may be associated with birth defect occurrence in newborns.

In addition to ancestry, most immigrant women in Taiwan form families with men of lower socio-economic status, and thus face multiple difficulties and challenges, including language, food, beliefs, culture, living habits, parenting, economic deprivation, and lack of social support [11,12]. Moreover, many of these environmental disadvantages are directly or indirectly correlated with birth defects in newborns [13,14,15]. For example, a long-term survey in Canada discovered that the birth defect rate in newborns of immigrant mothers from less developed countries (Sudan, Jamaica, Bangladesh, and Afghanistan) was 12–27% higher than that of non-immigrant mothers [16]. In the Netherlands, newborns of immigrant women from countries around the Mediterranean (Turkey and Morocco) had a 20% higher risk of birth defects than that of Dutch women [17].

However, it is worth mentioning that immigrant women are a highly select group who demonstrate strong resilience [18]. On average, they are physically and mentally healthier than Taiwanese women. Therefore, the prevalence of birth defects in newborns may be low. Some studies in the United States have supported this viewpoint and suggest that Hispanic mothers born abroad have a lower chance of giving birth to children with birth defects [19,20]. This phenomenon may be exemplified by the “healthy immigration effect” [18]. However, there are not yet data in Taiwan to verify this possibility. This study uses 10 years of data from the National Health Insurance program and the entire population in Taiwan as the research subjects. By controlling for the variables of mothers’ basic demographics and comorbidities, we compared the prevalence of birth defects from nine categories in newborns born to immigrant women and native-born women. Additionally, relevant demographic and social factors were investigated.

## 2. Methods

### 2.1. Data Collection

In this study, the data of newborns and their mothers were concatenated and analyzed from four national population databases between 2005 and 2014 in Taiwan. The databases include the National Health Insurance program in Taiwan, birth certificate applications, the birth certificate registry, and cause of death data. The health insurance database is part of the National Health Insurance program, which is a compulsory health care system that launched in 1995. The program has a coverage rate of more than 99% of Taiwan’s total 23 million population, making the information from the health insurance database representative for medical and public health research. The birth certificate application covers all live births in Taiwan (regardless of nationality) or stillbirths of 20 weeks and older and/or birth weight of 500 g and more. The statistical items include maternal background (nationality, place of residence), pregnancy conditions (risk factors or special treatment), spousal data (nationality, domicile), newborn data (birth weight, live birth/stillbirth, if a birth defect was noted, etc.) and other variables. The birth certificate registry information was obtained from the Ministry of the Interior of Taiwan. The data used contained basic demographics including ID number, gender, date of birth, gravidity and parity, place of birth (city/county), gestational age, birth weight, and location of birth (hospital/clinic/other). Additionally, parents’ ID numbers, education level, marital status, occupation, etc., were also included. The cause of death data compiled information from the cause written in the database or on the death certificate of Taiwanese nationals with a registered household. These data included the ID number, date of birth, date of death, place of death, location of death, and any details recorded by the International Classification of Disease (ICD) code on the death certificate.

**Figure 1:**
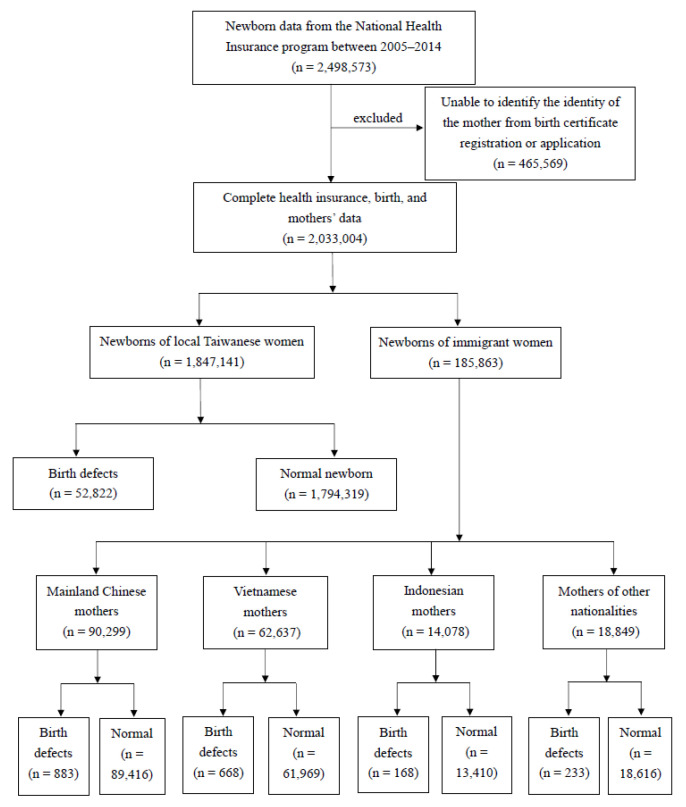
Sampling and grouping processes.

This study used data from 2005 to 2014 based on the following two considerations. (1) Prior to 2005, there were a considerable number of people listed as marriage-based immigrants but were in fact illegal work immigrants. This increased the complexity of the data cleaning process and may expand the number of samples and dilute the estimated disease rate. (2) After 2014, the number of immigrant women in Taiwan dropped significantly, and the number of children born also fell sharply. Because birth defects have a relatively low prevalence rate, an insufficient number of newborns made the estimation of the disease rate unstable. Therefore, this study stratified independent experimental groups using the top three countries of origin for marriage immigrants, which are mainland China, Vietnam, and Indonesia. New immigrants from other countries of origin are merged as “Others”, making up a total of four different groups of new immigrant women. The difference in birth defects were assessed through a comparison to newborns of native-born Taiwanese mothers. The detailed sampling and grouping processes are shown in Figure 1. Briefly, the birth certificate registry and applications were used to exclude mothers who could not be identified (*n* = 465,569). Those with complete data (*n* = 2,033,004) were divided by maternal nationality of origin and further categorized into groups with and without birth defects.

### 2.2. Categories of Birth Defect Diagnoses in Newborns

Based on diagnoses using the ICD system, this study classified birth defects into nine categories as the research outcome. The nine categories were the nervous system (including anencephaly, encephalocele, myelomeningocele, spina bifida, congenital hydrocephalus, microcephaly, holoprosencephaly, and cystic lymphangioma); the eye, ears, face and neck (including congenital cataract, microphthalmos and anophthalmos, congenital malformations of ear, anotia, and aicrotia); cleft lips and cleft palates (including cleft lip with/without cleft palate, cleft palate alone); the circulatory system (including atrial septal defect, patent ductus arteriosus, Tetralogy of Fallot, endocardium cushion defect, transposition of the great vessels, pulmonary valve atresia and stenosis, complex congenital heart disease, hypoplastic left heart syndrome, coarctation of the aorta, tricuspid valve atresia and stenosis, aortic valve stenosis, total anomalous pulmonary venous connection, single ventricle interrupted aortic arch, and double outlet right ventricle); the respiratory system (including choanal atresia, paralysis of vocal cords, congenital laryngomalacia, tracheomalacia, congenital cystic lung, congenital cystic adenomatoid malformation, pulmonary sequestration, and agenesis of the lung); the digestive system (including esophageal atresia/tracheoesophageal fistula, Hirschsprung disease, rectal and large intestinal atresia/stenosis, small intestinal atresia/stenosis, congenital pyloric stenosis, biliary atresia, and choledochal cysts); the genital and urinary organ systems (including renal agenesis/hypoplasia, urinary obstruction, hypospadias, indeterminate sex and pseudohermaphroditism, polycystic kidney, undescended and retractile testicle, epispadias, cystic kidney, bladder exstrophy, and cloacal exstrophy); the musculoskeletal system (including anomalies of the abdominal wall, diaphragmatic hernia, congenital deformities of feet, congenital dislocation of hip, polydactyly, syndactyly, limb reduction defects, dwarfism, inguinal hernia, congenital anomalies of skin, and clubfoot); and chromosomal abnormalities (Including: trisomy 21, trisomy 13, trisomy 18, Turner syndrome, XXY, and XXY)

### 2.3. Statistical Analysis

In this study, the prevalence of birth defects in newborns was calculated based on the number of birth defects and the total number of newborns in the four different ancestry groups. The prevalence of defects was calculated using the method recommended by the European Surveillance of Congenital Anomalies (EUROCAT) shown as the following. Total prevalence = total number of cases (live births, stillbirths, and medical interruptions of pregnancy) divided by the total number of births (live births and stillbirths). The chi-square test was used to compare the prevalence of birth defects between Taiwanese newborns and the newborns of immigrant mothers. In order to avoid possible confounding variables, we further used a multivariate logistic regression analysis to control for sociodemographic and clinical characteristics and compare the adjusted odds ratios of various defect categories between newborns of immigrant mothers and newborns of native-born mothers. The above analysis was performed using the SAS software (version 9.4) and *p* < 0.05 was considered to be statistically significant.

## 3. Results

The basic demographic distributions from the birth certificate registry (Table 1) showed that the stillbirth rate of newborns was about 1% in mothers of different nationalities. The stillbirth rate was higher for males (51.81–52.57%) and for single births (96.54–98.24%). The ages of mothers were mostly between 20 and 34 years old, the gestational ages were 37–40 weeks, and vaginal delivery was the main childbirth delivery method. The fetus weights were mostly between 2500 and 3499 g, and the birth location was mainly in hospitals, followed by clinics. About 6% of mothers of all nationalities suffered from genitourinary infections. Gestational diabetes mellitus (GDM) occurred more in native-born Taiwanese mothers (11.79%).

The percentages of newborns with birth defects from 2005 to 2014 categorized by the mother’s country of origin are shown in Table 2. From Table 2, it is apparent that the proportion of newborns with birth defects from mothers with various nationalities did not change much during these 10 years. However, the percentage of birth defects in every year (about 1.00%) for newborns born to non-Taiwanese mothers was significantly lower than the percentage of birth defects in newborns born to Taiwanese mothers (about 2.86%; *p* < 0.01). This result indicates that the prevalence rate of birth defects in newborns of immigrant women was significantly lower than that of newborns of native-born women. Additionally, the rates of birth defects in newborns within each nationality group were not significantly different during the 10 years. Therefore, the subsequent analysis used merged data from all 10 years.

Table 3 lists the prevalence of birth defects in newborns of mothers with varying nationalities from 2005 to 2014 according to different diagnostic categories. The prevalence of various types of birth defects was higher in newborns of Taiwanese mothers and was significantly higher than the prevalence rate in newborns of mothers of other nationalities (*p* < 0.001). Among children born to Taiwanese mothers, defects in the circulatory system were the most prevalent (0.92%). Similarly, heart defects were the most prevalent in children born to mothers of “other” nationalities (0.27%). However, the most common type of defects in children born to mothers from Mainland China, Vietnam, and Indonesia were those of the musculoskeletal system (0.24–0.42%). Children born to mothers of native nationality had the lowest rate of defects in the eye, ear, face, and neck (0.04–0.08%), and the children born to mothers of Vietnamese mothers had the lowest prevalence rate of defects in the respiratory system (0.03%).

In order to examine the influence of the age of the mothers on birth defects of newborns, Table 4 divides the mothers into two groups by age, ≤34 years old and ≥35 years old, and the prevalence of birth defects is shown. Table 4 demonstrates that children born to mothers of native mothers over 35 years old had a significantly higher rate of birth defects (1.29–3.27%) compared to children born to mothers under 34 years old (0.93–2.76%).

When controlling for live/still birth, fetal gender, birth year, number of births, birth location, type of delivery method, residing county/city, maternal age, gestational age, birth weight, and maternal comorbidities, the odds ratios of various birth defects in newborns of non-native mothers were all lower than newborns of local mothers (Table 5). The adjusted odds ratios (AORs) of the respiratory system (AORs ranged from 0.17 to 0.39) and the cardiac system (AORs ranged from 0.25 to 0.32) of newborns born to all immigrant mothers were the two lowest of all diagnoses. Moreover, the rates of chromosomal abnormalities (AORs ranged from 0.90 to 0.95) and cleft lip and palate (AORs ranged from 0.74 to 1.00) were not significantly different from newborns born to native-born mothers. Nervous system defects of newborns born to Indonesian mothers and other nationalities were the same as those born to Taiwanese mothers (AORs ranged from 0.65 to 0.71). The prevalence rate of defects in the eyes, ears, face, and neck in newborns born to Vietnamese and Indonesian mothers showed no difference from newborns of native-born mothers (AORs ranged from 0.54 to 0.81).

To explore the interaction with immigration status, stratification analyses by maternal age (≥35 years vs. ≤34 years) and DM status (disease vs. non-disease) were carried out in nine categories of birth defects. We found that newborns born to mothers over 35 years old had significantly higher risks of birth defects in the respiratory system (AORs = 0.34), musculoskeletal system (AORs= 0.54) and chromosomal abnormalities (AORs = 1.61) as compared to those born to mothers under 34 years old (AORs = 0.26, 0.53, and 0.70, respectively). Similarly, newborns of mothers with diabetes had higher risks of any birth defects (AORs = 0.43), cleft lips and cleft palates (AORs = 0.96), defects in the circulatory system (AORs = 0.33), those in the respiratory system (AORs = 0.40), the genital, urinary organs (AORs = 0.39) and the musculoskeletal system (AORs = 0.76) as compared to those of non-diabetes (AORs = 0.40, 0.93, 0.27, 0.27, 0.37, and 0.51, respectively).

## 4. Discussion

The results of this study show that regardless of nationality, the prevalence of various birth defects in newborns of immigrant women was lower than that of native-born women. This is consistent with the results of a previous study by Yang et al. [21] suggesting that the health status of the newborns of immigrant women was not any worse than their local counterparts in terms of stillbirth, underweight, birth defects, premature birth, or Apgar score, and three indicators—stillbirth, underweight and premature delivery—were better. Moreover, our results demonstrate that skeletomuscular system defects are the most common among newborns of women from the main immigrant countries, while circulatory system defects were the most common among newborns of Taiwanese women.

Congenital heart defects in newborns are the main problem related to birth defects worldwide. The European Surveillance of Congenital Anomalies (EUROCAT) stated that the overall birth defect rate of newborns from 2013 to 2019 was 22.6 (per 1000 births), and circulatory defects accounted for the majority, at a rate of 7.2, and while the rate was 2.0 for nervous system defects, 3.3 for limb defects, and 3.1 for urinary system defects [22]. Data from Asian countries showed that the overall defect rate was highest in Thailand, at 7.1 defects per 1000 births. The congenital heart defect rate was 1.9 in Thailand, 1.6 in China, 0.4 in Cambodia, 0.3 in Vietnam, and 0.1 in the Philippines [23]. However, these data in general were far lower than the defect rate seen in Taiwanese newborns, and even lower than some of the immigrant second-generation data. This may be attributed to the fact that the results of this study came from a nationally registered database, and the data on birth defects are more complete than other studies.

Birth defects cover a variety of genetic and environmental factors [24,25,26]. However, genetic mutation only causes a few specific diseases. Therefore, the phenomenon of an overall increasing prevalence of various defects would be more reasonable to consider from the perspective of differences in environmental conditions [27]. The conditions include socio-economic status, housing quality, maternal sociodemographic characteristics, and immigration status [28,29]. Thus, the phenomenon of birth defects in newborns of Taiwanese women being generally higher than those of the immigrant second generation may be attributed to the fact that the age of Taiwanese mothers was significantly higher than that of immigrant women. The high age at giving birth seems to be a common factor for all newborn defects [30,31]. The average prevalence of birth defects in offspring of mothers over 35 years old is high [32]. The childbearing age of Taiwanese women has gradually increased in the past few years. The average age of domestic women giving birth to their first child has reached 31 years old. Among them, the women over 35 years old accounted for 23%, which was 2.4 times higher than 10 years ago [33]. On the contrary, the childbearing age of immigrant women was mostly between 30 and 34 years old. Moreover, mothers under 20 years old accounted for 2.39% in Vietnamese women, and 2.38% in Cambodian women [5]. In this study, the percentage of childbearing Taiwanese mothers over 35 years old was close to 20%, while only 6–12% of immigrant women from the three main countries were over 35. This demonstrated that the childbearing age of native-born women was significantly higher than that of immigrant women, thereby increasing the prevalence of birth defects. In addition, many studies have indicated that immigrants are a highly select group, both physically and psychologically, and generally demonstrated more resilience [18]. These relatively healthy women may also give birth to healthier children compared to average women.

From the perspective of maternal physiological conditions, it is not difficult to understand why the prevalence of circulatory system defects in Taiwanese newborns was higher than that of all second-generation immigrants. Maternal chronic diseases (diabetes, hypertension, cardiovascular diseases, etc.) have been found to be associated with birth defects from 10 years of data in Taiwan [34]. Moreover, gestational diabetes is correlated with the occurrence of neonatal circulatory system defects [35]. Mothers with type 1 and type 2 diabetes before pregnancy increases the risk of neonatal circulatory system defects by about 4 times [36]. In this study, the prevalence rate of various diabetes in Taiwanese mothers was as high as 12%, which was much higher than that of immigrant women, which was less than 7%. In particular, the difference in gestational diabetes mellitus (GDM) was much more significant, probably due to the traditional postpartum confinement in Chinese society which gives Taiwanese women a higher probability of overnutrition. GDM is mainly caused by insufficient secretion of insulin or insulin resistance, and these reasons are currently considered to be related to genetic and environmental factors [37], such as high childbearing age, family history, or being overweight or obese [38], or specific ethnicities [39], among other factors. In recent years, the incidence of type 2 diabetes in Taiwan has also been rising, and the age of onset tends to get younger [40]. More expectant mothers may have been prediabetic or have type 2 diabetes before pregnancy [41]. Besides genetic factors, type 2 diabetes is also associated with obesity and obesity-prone lifestyles [42], which means that both genetic and environmental factors may indirectly cause heart defects in Taiwanese newborns.

Socio-economic status has always been considered to be related to birth defects. Pregnant women with lower family incomes, lower education levels, or who are unemployed have been linked to a higher risk of coronary heart disease (CHD) in general [43]. However, economic conditions may also influence the accessibility of medical resources and alter the estimated disease rates. Therefore, the prevalence of congenital heart disease in live births of Taiwanese newborns in this study was as high as 9.2 per 1000 births, which was significantly higher than every group of second-generation immigrants. We cannot rule out that ultrasound examinations were widely used among Taiwanese mothers (especially high-level ultrasounds not covered by insurance). This may lead to the early detection of fetal congenital heart disease and over-diagnosis, especially for patent ductus arteriosus (PDA) and atrial septal defect (ASDII) [44]. As economic status is generally poorer in immigrant families than in Taiwanese families [45], screening items not covered by insurance may not be affordable during pregnancy. Consequently, congenital heart diseases without obvious symptoms may not be identified in time. Therefore, the prevalence of circulatory system defects in Taiwanese newborns was higher than that of the second-generation immigrants. This may be due to differences in socio-economic status, thereby affecting the accessibility of health insurance services and contributing to over-diagnosis. However, due to a lack of individual-level indicators of socio-economic status in the database, whether healthier but poorer immigrants have a lower prevalence of birth defects warrants further investigation.

The lack of data on the length of residency of the immigrants in this study is another consideration in evaluating the environmental effect on congenital defects. Although the longer the new residents stay, the fewer cultural adaptation problems they experience [46], immigrant health declines with longer residence in the host country due to the loss of protective sociocultural factors [47] or marital maladjustment [48] according to the negative acculturation theory, which may be harmful to newborns’ health. For example, recent immigrants reported better health conditions than long-term immigrants [49] and had a lower risk of preterm birth [50]. It is not clear whether the lower prevalence rates of birth defects among newborns of immigrant mothers are invoked to a shorter duration exposed to the environment of Taiwan in immigrant women than local ones in this study. Nevertheless, this is an important issue to be addressed in future research while the information of length of residency of the immigrants is available.

## 5. Conclusions

This article used the National Health Insurance database and other connecting databases to obtain data from 2005 to 2014 to examine the differences in birth defect rates in newborns of mothers of different nationalities. When controlling for other risk factors of birth defects, newborns of immigrant women, regardless of their nationality, showed a lower prevalence rate of various birth defects compared to newborns of native-born women. It is obvious that immigrants are a highly select group. With the influence of the healthy immigration effect, their children are heathier than those of the locals. On the contrary, high childbearing age and type 2 diabetes led to a higher rate of birth defects in native-born Taiwanese women, who are also participating in higher education and delaying marriage. Furthermore, over-diagnosis caused by accessibility to health insurance services associated with socio-economic status may also be one of the reasons that a high prevalence of birth defects was observed in Taiwanese newborns. These results refute the stereotypes that the second-generation immigrants have a higher birth defect rate and poorer health conditions and emphasize the importance of appropriate childbearing age, control of chronic diseases during pregnancy (especially diabetes), and socio-economic conditions to newborn health.

## Figures and Tables

**Table 1:** Basic demographic distribution of newborns registered between 2005 and 2014, stratified by maternal origin.

	Nationality									
	Taiwan (*n* = 1,847,141)		Mainland China (*n* = 90,299)		Vietnam (*n* = 62,637)		Indonesia (*n* = 14,078)		Others (*n* = 18,849)	
	*n*	%	*n*	%	*n*	%	*n*	%	*n*	%
Live births and fetal deaths										
Live births	1,826,327	98.88	89,655	99.29	62,164	99.22	13,944	99.01	18,632	98.83
Stillbirths	20,814	1.13	644	0.72	473	0.78	134	1.00	217	1.17
Fetal gender										
Male	960,578	52.01	47,318	52.41	32,757	52.12	7397	52.57	9760	51.81
Female	885,907	47.96	42,956	47.57	29,867	47.87	6676	47.39	9086	48.18
Unknown	656	0.04	25	0.03	13	0.02	5	0.03	3	0.02
Single or multiple births										
Single	1,789,370	96.88	87,215	96.54	61,467	98.07	13,827	98.24	18,279	96.94
Multiple	57,771	3.12	3084	3.46	1170	1.94	251	1.77	570	3.06
Birth location										
Hospital	1,303,655	70.56	61,039	67.78	36,010	58.31	8140	58.44	13,093	69.76
Clinic	540,754	29.30	29,178	32.13	26,536	41.56	5843	40.83	5704	29.97
Other	2732	0.15	82	0.09	91	0.14	95	0.74	52	0.28
Gestational age										
<24 weeks	13,477	0.73	346	0.39	245	0.42	62	0.49	115	0.63
24–36 weeks	171,636	9.29	6,193	6.90	4599	7.57	1061	7.58	1615	8.57
37–40 weeks	1,607,241	87.01	79,287	87.85	55,136	88.08	12,313	87.49	16,298	86.47
≥41 weeks	54,787	2.98	4473	4.87	2657	3.95	642	4.46	821	4.33
Age of mother										
<20	27,112	1.48	25	0.03	1,52	1.58	233	1.56	68	0.36
20–34	1,452,836	78.74	79,222	87.47	58,204	92.04	12,300	86.21	14,310	75.48
≥35	367,193	19.79	11,052	12.51	3181	6.39	1545	12.23	4471	24.17
Maternal diseases (from health insurance data)										
Hypertension	41,379	2.23	1698	1.89	1101	1.74	272	1.97	354	1.88
Cardiovascular disease	4041	0.22	328	0.37	165	0.28	52	0.38	58	0.31
Renal disease	6515	0.36	308	0.35	205	0.33	45	0.33	88	0.47
Genitourinary infection	116,062	6.27	5936	6.59	4111	6.64	870	6.29	1212	6.48
General infection	23,145	1.26	1211	1.35	869	1.41	193	1.34	248	1.32
Anemia	104,624	5.64	4339	4.81	3068	4.99	723	5.25	872	4.68
Drug abuse/dependence	4407	0.24	446	0.49	265	0.40	64	0.45	69	0.37
Mental disorder	48,452	2.62	3770	4.19	2494	3.88	557	3.98	725	3.87
Alcohol-related conditions	871	0.05	103	0.12	46	0.06	14	0.10	18	0.10
DM										
GDM	218,327	11.79	6153	6.90	3783	6.77	680	5.19	1434	7.73
Type 2 DM (within 2 years)	8764	0.47	203	0.23	127	0.22	29	0.22	68	0.37
Type 2 DM (2–5 years)	13,193	0.71	208	0.24	112	0.24	33	0.27	70	0.39

**Table 2:** Number and percentage of newborns with birth defects, stratified by maternal origin.

Year	Nationality of Mother														
	Taiwan			Mainland China			Vietnam			Indonesia			Others		
	Total Births	Number of Newborns with Birth Defects	%	Total Births	Number of Newborns with Birth Defects	%	Total Births	Number of Newborns with Birth Defects	%	Total Births	Number of Newborns with Birth Defects	%	Total Births	Number of Newborns with Birth Defects	%
2005	181,326	5298	2.92	10,169	124	1.22	12,985	148	1.14	2301	25	1.09	2299	26	1.13
2006	182,361	5449	2.99	10,426	99	0.95	10,211	105	1.03	1948	18	0.92	2296	31	1.35
2007	183,992	5453	2.96	10,032	89	0.89	7805	84	1.08	1732	27	1.56	2007	23	1.15
2008	179,013	5290	2.96	9607	94	0.98	6559	57	0.87	1488	13	0.87	1885	19	1.01
2009	177,425	5220	2.94	8754	65	0.74	5380	58	1.08	1281	16	1.25	1735	26	1.50
2010	153,795	4379	2.85	7921	78	0.98	4094	39	0.95	1122	17	1.52	1575	13	0.83
2011	185,255	5174	2.79	8608	82	0.95	4134	42	1.02	1085	7	0.65	1626	23	1.41
2012	219,656	5616	2.56	9654	109	1.13	4618	46	1.00	1186	17	1.43	1929	24	1.24
2013	183,919	5340	2.90	7453	68	0.91	3456	48	1.39	992	17	1.71	1675	31	1.85
2014	200,399	5603	2.80	7675	75	0.98	3395	41	1.21	943	11	1.17	1822	17	0.93
Total	1,847,141	52,822	2.86	90,299	883	0.98	62,637	668	1.07	14,078	168	1.19	18,849	233	1.24

**Table 3:** Distribution of birth defects in newborns in Taiwan, stratified by maternal origin.

Category	Taiwan		Mainland China		Vietnam		Indonesia		Others	
	Number of Newborns with Defects	Prevalence Rate (%)	Number of Newborns with Defects	Prevalence Rate (%)	Number of Newborns with Defects	Prevalence Rate (%)	Number of Newborns with Defects	Prevalence Rate (%)	Number of Newborns with Defects	Prevalence Rate (%)
Nervous system	2850	0.15	49	0.05 *	49	0.08 *	12	0.09 *	20	0.11
Eyes, ears, face, and neck	1535	0.08	32	0.04 *	22	0.04 *	6	0.04	13	0.07
Cleft lip and cleft palate	3582	0.19	123	0.14 *	113	0.18	22	0.16	26	0.14
Circulatory system	16,922	0.92	136	0.15 *	125	0.20 *	36	0.26 *	50	0.27 *
Respiratory system	5261	0.28	46	0.05 *	38	0.03 *	15	0.1 1*	9	0.05 *
Digestive system	5811	0.31	96	0.11 *	69	0.11 *	17	0.12 *	30	0.16 *
Genital, urinary organs	10,021	0.54	163	0.18 *	103	0.16 *	29	0.21 *	33	0.18 *
Musculoskeletal system	8758	0.47	214	0.24 *	148	0.24 *	59	0.42	46	0.24 *
Chromosomal abnormalities	2769	0.15	80	0.09 *	48	0.05 *	13	0.09	29	0.15

* p < 0.001 as compared with the rate of Taiwanese newborns.

**Table 4:** Cross-distribution of maternal origin and age of mother considering newborns with birth defects.

Age of Mother	Taiwan			Mainland China			Vietnam			Indonesia			Others		
	Total Births	Number of Newborns with Defects	%	Total Birth	Number of Newborns with Defects	%	Total Birth	Number of Newborns with Defects	%	Total Birth	Number of Newborns with Defects	%	Total Birth	Number of Newborns with Defects	%
≤34 years old	1,479,948	40,804	2.76	79,247	734	0.93	59,456	627	1.05	12,533	140	1.12	14,379	171	1.19
≥35 years old	367,193	12,018	3.27	1052	149	1.35	3181	41	1.29	1545	28	1.81	4470	62	1.39

**Table 5:** Adjusted odds ratios of birth defects in newborns of immigrant mothers as compared with native Taiwan-born mothers using multiple logistic regression analyses.

	Adjusted Odds Ratios ^§^				
	Taiwan	Mainland China	Vietnam	Indonesia	Others
All defects	Reference	0.37 (0.35–0.39)	0.41 (0.38–0.44)	0.47 (0.40–0.54)	0.43 (0.38–0.49)
Nervous system	Reference	0.43 (0.32–0.57)	0.58 (0.43–0.77)	0.65 (0.37–1.15)	0.71 (0.46–1.10)
Eyes, ears, face, neck system	Reference	0.45 (0.30–0.69)	0.54 (0.24–1.21)	0.81 (0.47–1.41)	0.45 (0.30–0.69)
Cleft lip and cleft palate	Reference	1.00 (0.83–1.21)	0.86 (0.56–1.31)	0.74 (0.50–1.08)	1.00 (0.83–1.21)
Circulatory system	Reference	0.25 (0.21–0.30)	0.32 (0.23–0.45)	0.30 (0.23–0.40)	0.25 (0.21–0.30)
Respiratory system	Reference	0.23 (0.17–0.31)	0.39 (0.23–0.65)	0.17 (0.09–0.32)	0.23 (0.17–0.31)
Digestive system	Reference	0.37 (0.29–0.46)	0.41 (0.25–0.66)	0.53 (0.37–0.76)	0.37 (0.29–0.46)
Genital, urinary organs	Reference	0.33 (0.28–0.41)	0.44 (0.30–0.63)	0.33 (0.23–0.47)	0.33 (0.28–0.41)
Musculoskeletal system	Reference	0.55 (0.47–0.65)	0.50 (0.34–0.72)	0.53 (0.39–0.70)	0.55 (0.47–0.65)
Chromosomal abnormalities	Reference	0.90 (0.67–1.20)	0.94 (0.54–1.65)	0.95 (0.64–1.41)	0.90 (0.67–1.20)

^§^ Controlling for live births/stillbirths, fetal gender, birth year (2005–2009 or 2010–2014), single/multiple births, birth location, type of delivery method, residing county/city, maternal age, gestational age, birth weight, and maternal comorbidities.

## Data Availability

The data utilized in this study are mainly from a part of the National Health Insurance Research Database (NHIRD) which is protected by the “Personal Information Protection Act” executed by Taiwan’s government. Links regarding contact info for which data requests may be sent to the following website: https://nhird.nhri.org.tw/.
